# Clairvoyance

**Published:** 1886-05

**Authors:** 


### HALL'S JOURNAL OF HEALTH

Vol. 33. MAY, 1886. No. 5.

**They are Coming**.—We desire to call special attention to the effort now being made to place Hall's Journal of Health in the lead of periodicals exclusively devoted to life and health, and we are glad to be able to assure our readers that our subscription list is rapidly filling up, but there is and always will be room for more. Our single and club rates are so moderate that we should not lack support from the masses for whose benefit we have labored for more than thirty-three years.

### CLAIRVOYANCE.

The term which heads this article has been adopted into our English tongue from the French. In its native vernacular it signifies physical clear-sightedness, but by common consent, justified by free usage, it has acquired a signification which leaves wholly out of view the merely *physical* and reaching quite over it, takes on the *spiritual*, thus recognizing the existence of an interior perception of remarkable clearness and delicacy, in no wise dependent upon the ordinary organs of sight.

The existence of this faculty in man has been too clearly demonstrated to be longer open to question as a fact. Indeed it is safe to affirm that it is inherent in all persons, and might be cultivated and developed to a point of great usefulness in very many instances, where it is now suffered to slumber without recognition.

As a class, Physicians have been altogether too backward in giving it place, as a valuable auxiliary to the healing art, notwithstanding the repeated proofs of its efficacy in this behalf.

The late Doctor Samuel B. Brittan, so well and widely known as a popular lecturer upon what are usually termed liberal subjects, often availed himself of the insight respecting the condition and needs of patients, which clairvoyants were able to give him, and his practice as a specialist in medicine, was greatly assisted in this way.

One in particular, whom he used to consult, was the now decease Mrs. Mettler, whose chief centres for consultation and advice, were the cities of New York and Hartford, to which, for a number of years, she paid alternate visits, with the single object of making available her clairvoyant powers in ministering to the afflicted.

I will relate a single instance, as related to me by Doctor Brittan, who in the course of a lecturing tour, was a guest at the house of a well known family then residing in Michigan, one of whose younger male members had been bed-ridden for a long period, from the effects of a hitherto incurable gun-shot wound.

The Doctor brought to New York a lock of hair of the sufferer, and placing it in Mrs. Mettler's hand, awaited the diagnosis.

It should be stated for the information of such of the Journal's readers as are unacquainted with the fact, that the lock of hair served to bring the patient and the clairvoyant *en rapport;* in other words, to establish such intelligibly harmonious conditions between them as to enable the mysterious faculty of the one to discern the physical needs of the other.

Mrs. Mettler gave an accurate description of the patient, affirming that he was prostrated by a severe gun shot wound in the thigh; giving also a minute account of the accident, and stating among other things, that the contents of the gun, on being discharged. passed through a trousers pocket, and carried a large copper cent into the wound, which obviously could not heal until the cent was removed.

This being communicated to the local medical attendant of the sufferer, was much derided.

He declared that he, who had attended the patient almost daily, and watched the progress of the wound from the beginning, was better able to give a correct diagnosis, than a person a thousand miles away, who had never seen it..

But was he correct in this opinion? Let us see what followed. A devoted sister had day after day and week after week dressed the obstinate wound, which showed no sign of healing. At length she observed that a hard substance had worked its way to the external opening, and by a dexterous use of a pair of pointed scissors she disengaged and took away an old-fashioned *copper cent.* After that the wound rapidly healed, and for aught we know to the contrary the patient is still alive and well.

Another remarkable instance of discovery and relief through the means of clairvoyance, occured in Brooklyn a few years ago. A young lad who had been carefully and tenderly brought up, was unaccountably and strangely afflicted.

He was able neither to eat, sleep, or rest, as healthy children do, and growing worse from day to day, became at length so emaciated and weak from long suffering that there remained little hope of his recovery.

A lady friend who paid the family a visit, told them of a renowned clairvoyant, of Providence, R. I., and as she was on the point of returning there, volunteered to take to her a lock of the child's hair, as a means of clairvoyantical diagnosis.

The information received was that the boy, whilst sojourning in the country the previous summer, had drank of a stream boy-fashion, and thus imbibed a water snake which had continued to live and grow in his stomach, until now it had so poisoned his entire system that it would be almost impossible to save the lad's life. She said, moreover, that she could so treat the child as to rid him of this fatal intruder, but that even then, it would almost surely follow that one so completely impregnated with poison would die. It was indeed, almost a "forlorn hope."

The members of the family were naturally enough skeptical of this diagnosis obtained through such incomprehensible means, but the insistance of the mother, who had for so long and tenderly watched over the fading image of her child, induced his removal to Providence, where he was placed under the treatment of the lady in question. The near result was the passage from the boy of the disumpted remains, in three parts, of a *water snake*, which I am informed, is still preserved by the family as indubitable proof of the facts above related.

I am sorry to add that the patient survived the treatment for only a brief period, his whole body becoming distinctly marked in spots from the unquestioned effects of the poisoning to which it had been subjected.

It may be aptly asked, why is it, if such things are true, that a means of cure so efficient is not generally acknowledged and more frequently resorted to?

I am only able to reply that it is considered by the medical profession generally, as quite irregular and unauthorized, and a thing to be put down rather than encouraged.

There are, however, some rare exceptions to this rule, but not always generous ones, frequent clairvoyantical diagnoes being made from locks of hair and other means of magnetic concurrence, at the instance of attending physicians, who purposely keep the medium in the background, and take all the credit of the cure.

But the days of an exclusive property in patients, on the part of the votaries of any recognized school of medicine, it is to be hoped, are numbered, and that the populace may be free to adopt such methods of cure as their judgment dictates, or even suffered, when the inexorable visitant knocks at the door, to die a *natural death*, despite of class legislation for the protection of any special profession or employment.

Common sense is a gift of the gods, which should not be left altogether out of view even by our law makers, and freedom for one's self and others in all private concerns should never be impugned.

